# MicroRNAs and Natural Compounds Mediated Regulation of TGF Signaling in Prostate Cancer

**DOI:** 10.3389/fphar.2020.613464

**Published:** 2021-01-27

**Authors:** Zeeshan Javed, Khushbukhat Khan, Amna Rasheed, Haleema Sadia, Shahid Raza, Bahare Salehi, William C. Cho, Javad Sharifi-Rad, Wojciech Koch, Wirginia Kukula-Koch, Anna Głowniak-Lipa, Paweł Helon

**Affiliations:** ^1^ Office for Research Innovation and Commercialization, Lahore Garrison University, Lahore, Pakistan; ^2^ Atta-ur-Rahman School of Applied Biosciences (ASAB), National University of Sciences and Technology (NUST), Islamabad, Pakistan; ^3^ School of Basic Medical Sciences, Lanzhou University, Lanzhou, China; ^4^ Department of Biotechnology, Balochistan University of Information Technology, Engineering and Management Sciences, Quetta, Pakistan; ^5^ Medical Ethics and Law Research Center, Shahid Beheshti University of Medical Sciences, Tehran, Iran; ^6^ Department of Clinical Oncology, Queen Elizabeth Hospital, Hong Kong, China; ^7^ Phytochemistry Research Center, Shahid Beheshti University of Medical Sciences, Tehran, Iran; ^8^ Facultad de Medicina, Universidad del Azuay, Cuenca, Ecuador; ^9^ Chair and Department of Food and Nutrition, Medical University of Lublin, Lublin, Poland; ^10^ Department of Pharmacognosy, Medical University of Lublin, Lublin, Poland; ^11^ Department of Cosmetology, University of Information Technology and Management in Rzeszów, Rzeszów, Poland; ^12^ Branch in Sandomierz, Jan Kochanowski University in Kielce, Sandomierz, Poland

**Keywords:** prostate cancer, miRNAs, transforming growth factor-beta signaling, natural compounds, therapeutics, biomarkers

## Abstract

Prostate cancer (PCa) is with rising incidence in male population globally. It is a complex anomaly orchestrated by a plethora of cellular processes. Transforming growth factor-beta (TGF-β) signaling is one of the key signaling pathways involved in the tumorigenesis of PCa. TGF-β signaling has a dual role in the PCa, making it difficult to find a suitable therapeutic option. MicroRNAs (miRNAs) mediated regulation of TGF-β signaling is responsible for the TGF-*ß* paradox. These are small molecules that modulate the expression of target genes and regulate cancer progression. Thus, miRNAs interaction with different signaling cascades is of great attention for devising new diagnostic and therapeutic options for PCa. Natural compounds have been extensively studied due to their high efficacy and low cytotoxicity. Here, we discuss the involvement of TGF-*ß* signaling in PCa with the interplay between miRNAs and TGF-β signaling and also review the role of natural compounds for the development of new therapeutics for PCa.

## Introduction

Prostate cancer (PCa) is the fifth leading cancer cause of death worldwide. The mortality associated with PCa is strongly related to age. The highest incidence of PCa is observed in men over age 65 years. The recent advances in the field of genetic technologies have enabled us to delineate the genomic complexity of PCa (Rawla, 2019). It is a multifaceted disease orchestrated by a plethora of both intrinsic and extrinsic factors (Prasad et al., 2020). The complex molecular landscape of PCa renders it to escape cellular defenses and continue proliferation. Tumor development and progression is regulated by a number of cellular signaling pathways (Javed et al., 2020). Transforming growth factor-beta (TGF-β) has emerged as an essential modulator and mediator of the key steps of development of cancer such as the epithelial–mesenchymal transition (EMT), migration, and invasion (Behbahani et al., 2017). The TGF-β has a dual role in cancer. It has recently been shown that the dual nature of TGF-β can be due to its interaction with microRNAs (miRNAs) (Hao et al., 2019). These are small noncoding RNA in 21–25 nt that are crucial for the plethora of cellular processes (Javed et al., 2015). TGF-β signaling has been reported to regulate the formation of microprocessor complex *via* recruitment of mother against decapentaplegic homolog (SMADs) and influence the processing of pri-miRNAs (Miscianinov et al., 2018). PCa is the second most prevalent cancer in men after lung cancer, at which the cancer biomarker, that is, prostate specific antigen (PSA), is most commonly used for the PCa diagnosis. It has recently come to limelight that TGF-β signaling has broader implications in PCa. Both *in vitro* and *in vivo* approaches have shown the importance of TGF-β signaling as a new therapeutic approach for PCa (Hamidi et al., 2017). It is known that traditional medicines have been employed for centuries to treat cancers (Li and Weng, 2017). Interestingly, in recent times, the natural products and their derivatives have shown a tremendous potential for the treatment of PCa. (Leichter et al., 2017; Li et al., 2018a; Lu et al., 2018). Here, we discuss the interaction between TGF-β signaling and miRNAs in PCa, and also review the importance of natural compounds as a new therapeutic intervention in PCa.

## PCa Treatment and Prognosis

PCa is an androgen-dependent cancer; it originates from the peripheral zone of the prostate. Alterations in the genetic framework of PCa lead to variations in the tumor physiology, including different tumor grades, aggressiveness of the tumor, and various treatment options (Hayes and Barry, 2014). Treatment options for PCs include prostatectomy, chemotherapy, hormonal therapy and radiation therapy. For low-volume and low-grade cancers, palliative therapy is also an option while androgen deprivation therapy (ADT) which includes the bilateral orchiectomy with androgen antagonist or agonist of GnRH is used for advanced grade prostate tumors. In high-grade metastatic prostate tumors, a combination of ADT and chemotherapy is used. Castration-resistant PCas involve different second-line treatments which include chemotherapy, antiandrogen therapy, steroid inhibitors, immunotherapy, and radioactive treatments (Mottet et al., 2017). Tumor grades and stage of disease determine the prognosis of PCa patients. Majority of the people suffering from PCa undergo organ-confined surgery and radiation therapy (Donovan et al., 2016). The survival frequency of PCa patients over the span of five years has been reported to be 27–53%. In patients with advanced grade prostate tumor, the survival and recurrence of diseases is measured by the relative decrease in PSA levels and response to ADT (Matulewicz et al., 2017).

## TGF-β Signaling in Cancer

The TGF-β superfamily involve in development and differentiation of various mammalian cells. It consists of a broad range of proteins, such as the bone morphogenetic proteins (BMPs), anti-muellerian hormones (AMH), activins, growth factors, differentiation factors, and isoforms of TGF-β (TGF-β1, TGF-β2, and TGF-β3) that act as regulators of diverse cellular processes. This superfamily has been reported to orchestrate the key cellular processes and interactions (development, differentiation, migration, invasion, apoptosis, and immune responses) (Nacif and Shaker, 2014). TGF-β signaling has been reported for the tissue homeostasis (Xu et al., 2018). The role of TGF-β in cancer is debatable as it can either be tumor suppressor or oncogenic (Drabsch and Ten Dijke, 2011). The complex molecular landscape of cells along with the varied extrinsic and intrinsic factors determines TGF-β role. During the early stages of development, the TGF-β signaling plays a constructive role and facilitates differentiation and development of cells; however, with the passage of time, TGF-β signaling plays a destructive role through promulgation of metastasis and invasiveness in various tissues. This complex behavior of TGF-β signaling has been referred to as TGF-β paradox (Tian and Schiemann, 2009; Gong et al., 2015). Abrogation in the TGF-β signaling cascade triggers the development of PCa (Barrett et al., 2017) during the later stages of life. This has led scientists to focus on the intricate behavior of TGF-β in the progression of PCa and other tumors. Recent technological advances enabled us to target TGF-β signaling for possible therapeutic intervention. Inhibiting TGF-β seems to be a promising strategy for the treatment of PCa.

**FIGURE 1:**
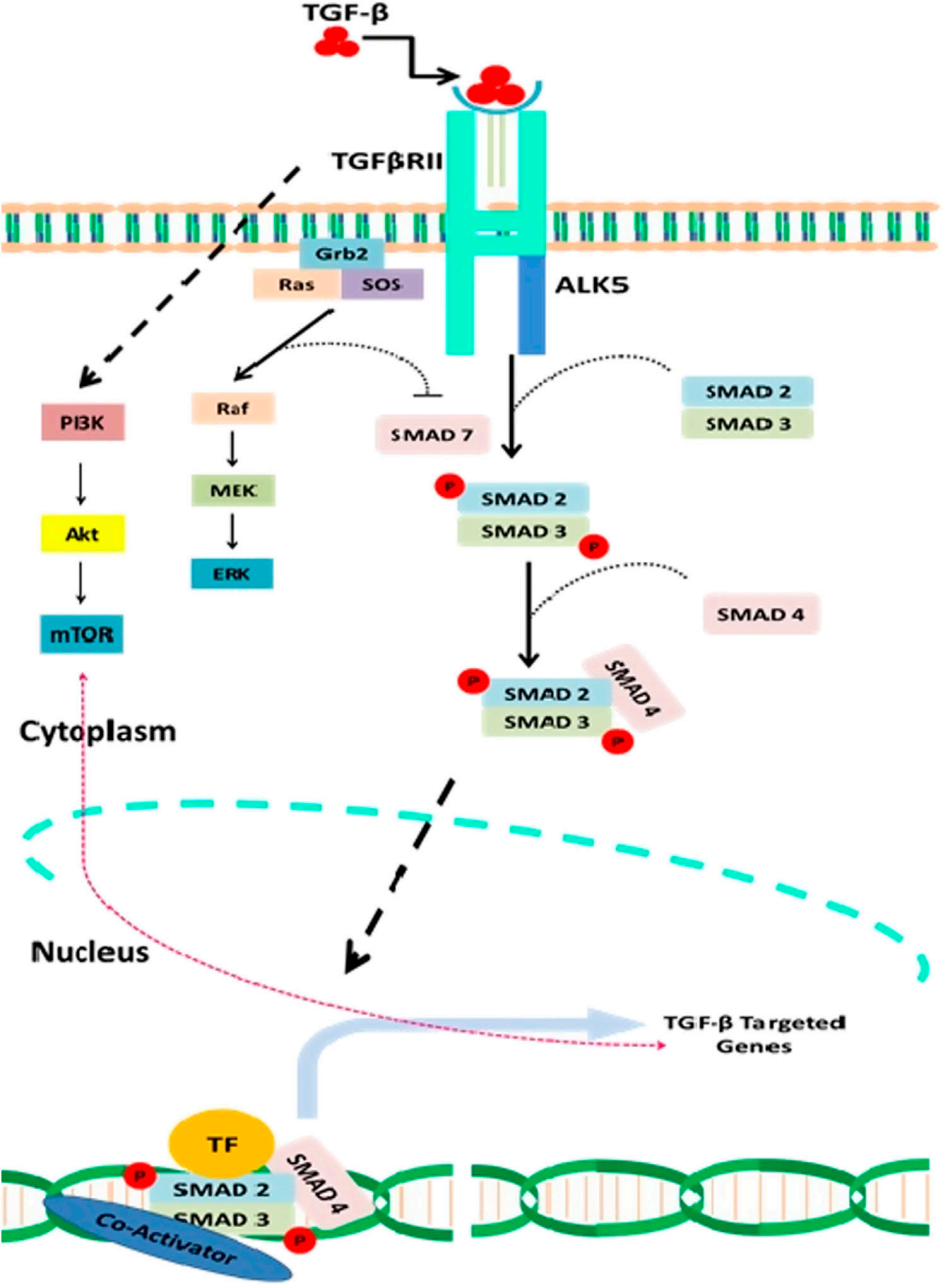
A detail description of both canonical and noncanonical TGF-β signaling and interaction of natural compounds in the regulation of this signaling cascade at various levels. Natural compounds such as the resveratrol modulate TGF-β pathway by inhibiting the receptor activity such as the attachment of TGF-β to TGF-βRII and thus prevent the downstream activation of Smads. Curcumin and Nobilitin both modulate Smad2/3/4 and prevent the activation of TGF-β pathway signaling genes such as the TWIST1, SNAIL, and SLUG. Caricoside E blocks the formation of Smad complex and their translocation to the nucleus. Arctigenin inhibits the TGF-β–mediated activation of ERK and thus triggers apoptosis. Baicalin has also been reported that inhibit TGF-βRII expression and thus downstream signaling of TGF-β pathway.

Three isoforms of TGF-β are having distinctive functions and play a crucial role in cellular growth (Akhurst, 2017). The latency associated protein (LAP) regulates the activation of TGF-β receptor through formation of latent TGF-β–binding protein (LTBP) (Nickel et al., 2018). The activated TGF-β then triggers either canonical or noncanonical TGF-β signaling, which involves activation of Smads (Smads 2,3 and 4) in case of canonical TGF-β signaling, while the non-canonical TGF-β signaling does not involve the activation of Smads (Massagué, 1998; Lodyga and Hinz, 2020). The noncanonical TGF-β signaling mechanistically promotes the activation of phosphatidylinositol-3-kinase/AKT/mammalian target of rapamycin (PI3K/AKT/mTOR), Janus kinase and p38 (JNK/P38), Rho-GTPase, and mitogen-activating protein kinases (MAPK).

The noncanonical signaling pathway is triggered by the same TβRI and TβRII complex that mechanistically promotes the activation of the broad range of molecular cascades such that the conjointly canonical and noncanonical signaling regulates a myriad of cellular functions ranging from posttranslational modifications to the binding of proteins to the target genes. TGF-β signaling plays a crucial role in the cellular development *via* inhibiting cellular growth. Loss of function mutations in TGF-β pathway has been reported to trigger uncontrolled cellular growth that ultimately leads to the development of tumor. Abnormal TGF-β signaling has been reported to accelerate carcinogenesis (Zhang et al., 2013). miRNAs contribute to the transcriptional activity of the TGF-β pathway, indicating functional links between short noncoding RNAs and TGF-β pathway. (Figure 1)

## Altered TGF-β Signaling in PCa

TGF-β signaling is responsible for growth, proliferation, differentiation, metastasis, invasion, and apoptosis of both stromal and epithelial cells of prostate tissue (Barrack, 1997). Alterations in TGF-β signaling result in the development of PCa. TGF-β1 has been documented to be overexpressed in PCa. There was an increased level of TGF-β1 protein in tissue, serum, and urine of PCa patients (Reis et al., 2011; Liu et al., 2014). TGF-β1 increased levels curtail the grade, stage, invasiveness, angiogenesis, and metastasis of PCa (Reis et al., 2011). In addition, TGF-β1 expression also correlates with the survival rates of the patients. Loss or downregulation of TGF-β receptors is the most frequent alteration observed in PCa. Nearly 30% of PCa have altered or downregulated the expression of TGF-β receptors (Yumoto et al., 2016). Furthermore, expression of TGFβR1 and TGFβR2 was low in PCa with metastatic potential as compared to localized primary tumors. TGFβR2 upregulation promotes apoptosis and inhibits metastasis in PCa cells *via* activation of caspase-1 (Pu et al., 2009). TGFβR2 activates the expression of TGF-β1, a precursor for the activation of caspase-mediated apoptosis. However, downregulation of TGFβR2 promotes malignant transformation in PCa cells (Pu et al., 2009). These findings suggest that TGFβR2 downregulation plays a pivotal role in the progression of resistant PCa cells. TGFβR2 has proven to be a tumor suppressor gene. Hypoxic activation of DNA methyltransferases is the key enzyme responsible for the downregulation of TGFβR2 in PCa cells. DNA methyltransferases triggers the hypermethylation of the promoter region of TGFβR2 which in turn inhibits gene activation (Brattain et al., 1996). Prior to this, it was reported that mutations in the promoter region of TGFβR2 led to the downregulation of this apoptosis-inducing gene. In addition to TGFβR2, downregulation of TGFβR3 is the most prevalent modification of the TGF-β cascade in PCa (Brattain et al., 1996). Downregulation of TGFβR3 promotes invasiveness, motility, and metastasis of PCa cells both *in vitro* and *in vivo* (Turley et al., 2007). It is reported that in normal prostate epithelial cells, downregulation of TGFβR3 expression resulted in the development of cancer stem cell phenotype and impeded cell to cell contact (Sharifi et al., 2007). Testosterone and dihydrotestosterone (DHT) are the activators of androgen receptor (AR) signaling. AR is a nuclear receptor whose activations result in its translocation to the nucleus where it modulates the expression of the target genes directly or indirectly. AR-mediated direct expression of target genes includes binding to AR–binding elements (AREs) and genes, while the indirect expression involves the regulation of various transcription factors. TGF-β signaling interacts with AR signaling and regulates it to certain extent. In PCa, the expression of TGF-β–targeted genes is influenced by AR signaling. SMAD3 interacts with AR and impedes SMAD3 binding to the SBEs (SMAD-binding elements) (Chipuk et al., 2002). DHT has been reported to inhibit the expression of TGFβR2. The DHT-mediated attenuation of the expression of TGFβR2 in turn decreases the binding of SP1 to the promoter genes. The downregulation of TGFβR2 promotes the apoptosis in prostate adenocarcinoma cells *via* upregulation of TGFβR2-targeted genes such as the cyclin Ds, Bcl-xL, and caspase-3 (Song et al., 2008). Abrogation in the AR signaling leads to cell survival, growth, and motility in PCa cells. The differentiation of the PCa cells is affected by the defected AR signaling which increases the overexpression of TGF-β and triggers growth, viability, aggressiveness, and invasiveness of the androgen-resistant PCa cells. TGF-β and AR synergistically stimulate apoptosis in PCa cells overexpressing TGFβR2 (Steiner and Barrack, 1992). The interplay between AR and SMAD4 proteins synergistically stimulates apoptosis in PCa cells with overexpressed TGFβR2 (Zhu and Kyprianou, 2010). It has been documented that administration of DHT to PC-3 cells, can lead to activation of EMT through interaction with SNAIL protein. The DHT administration increases the expression of N-cadherin which in turn inhibits the expression of E-cadherin and beta catenin and stimulates the activation of EMT. In addition, TGF-β signaling interacts with AR signaling pathway to facilitate the expression of TWIST1 that triggers the activation of EMT in PCa cells (Shiota et al., 2012). These findings indicate that TGF-β signaling has a decisive role in promoting invasiveness of PCa cells. The tumor cells employ a vast range of strategies to escape apoptosis and retain progressive growth and invasiveness (Hu et al., 2014). The tumorigenic prostate epithelial cells escape the apoptotic TGF-β signaling *via* constitutive activation of Akt pathway. The activated Akt pathway prevents the nuclear translocation of TGF-β–regulated Smad3 and arrest the growth of proteins such as the p21. The PI3K/Akt/mTOR pathway activates EMT in PCa cells through modulating the expression of TGF-β (Ao et al., 2006). Downregulation of PI3K/Akt/mTOR results in the inhibition of TGF-β–induced expression of vimentin. These in turn promote the downregulation of keratin and thus increase invasiveness of the tumor cells. Nuclear factor-kappa B (NF-κβ) has also been reported to activate EMT in PCa cells through its interplay with TGF-β signaling. It has been reported that overexpression of NF-κβ is modulated by the overexpression of TGF-β. The NF-κβ expression elevates the synthesis of vimentin and increases metastasis and invasion in PCa cells (Ao et al., 2006). Inhibition of either TGF-β or NF-κβ suppressed the invasion of cancer cells and the EMT process.

## Interplay Between TGF-β Pathway and miRNAs in PCa

miRNAs form a class of endogenous, small (19–25 nucleotides), single-stranded, noncoding RNA molecules (Finotti et al., 2019), which progressively contribute to a vast range of critically important biological events such as development (Wienholds and Plasterk, 2005; Cho et al., 2019), proliferation (Zhuang et al., 2018), differentiation (Li et al., 2018b), apoptosis (Slattery et al., 2018), signal transduction (Barbu et al., 2020), and many more. Expression patterns of miRNAs are linked with a wide range of anomalies; thus, screening and characterization of miRNAs can serve as a potential diagnostic and therapeutic tool (Dwivedi et al., 2019). Till date, more than 25,000 miRNA sequences have been identified, and this number is expected to grow. According to an estimation, 3–4% of human genome comprises miRNAs (Valinezhad Orang et al., 2014). These miRNAs interfere with numerous key regulators of cellular processes by binding with posttranscriptional products. For this reason, miRNAs are considered as important biomarkers for many cancers, including PCa. Here, we shall focus on the interplay between various miRNAs and TGF-β signaling regulators with a focus on PCa.

Growing bodies of evidence have revealed multiple miRNAs–TGF-β checkpoints that control TGF-β signaling in different manners and intrinsically control the progression of PCa. For instance, SMAD family appeared to be the major target of miRNAs. The miRNAs can affect progression of PCa in individual or in combination with other miRNAs. Moreover, many miRNAs may interact in direct or indirect manners. Overexpression of miR-486-5p downregulates the expression of SMAD2 to promote cell pre-filtration in PCa which was reversed by knocking miR-486-5p (Yang et al., 2017). Another miRNA named miR-505-3p has the ability to interact with both SMAD2 and SMAD3 to contribute in PCa progression (Tang et al., 2019). MiR-19a-3p is a tumor suppressor miRNA that targets the SMAD2 and SMAD4 resulting in inactivation of TGF-β and suppression of PCa (Wa et al., 2018). Interactions of miRNAs with TGF-β pathways also indirectly controlled by other key players as well. For instance, TR4, a transcription regulator, can suppress the expression of miR-373-3p which otherwise would inhibite SMAD3 through inhibition of TGF-βR2 (Qiu et al., 2015). Interestingly, miRNAs can work in clusters to regulate tumor progression as well. For instance, a cluster of miR-15a/16 controls TGF-β signaling by downregulation of *p*-SMAD3, ACVR2A, Snail, and Twist, resulting in attenuated expression of TGF-β–dependent genes MMP2 and E-cadherin. This condition leads to the inhibition of EMT and invasion of PCa in LNCaP cells (Jin et al., 2018). Similarly, another miRNA cluster i.e., miR-122/132 downregulates SOX4 and disrupts the EMT process to suppress PCa (Fu et al., 2016). On the other hand, single miRNAs are also capable of controlling progression of PCa by interacting with TGF-*ß*. For example, miR-34 interacts with TGF-*ß* signaling through SMAD3 and suppresses PCa (Fang et al., 2017). Just like SMAD3, SMAD4 is also a vital target of many miRNAs. A study has shown that overexpression of miR-1260b suppressed SMAD4 and promoted PC progression, whereas genistein-induced downregulation of miR-120b resulted in increased expression of SMAD4 and sFRP1, hence promoting apoptosis (Hirata et al., 2014b). Hyperglycemia-induced overexpression of miR-301a also suppresses p21 and SMAD4 which result in G1/S cell cycle transition and cell proliferation ultimately (Li et al., 2018c). Another miR-205 targets 3′UTR of SMAD4 and downregulates its expression to promote PCa (Zeng et al., 2016).

Apart from the SMAD family, miRNAs also interact with TGF-β receptors. Hypoxia-induced elevated levels of miR-93 promote PCa through degradation of TGFβR2 (Zhou et al., 2018). miR-133b plays its role in the suppression of PCa by downregulation of TGFβR1 and TGFβR2. Attenuated expressions of miR-133b lead to activation of TGF-β signaling and progression of PCa (Huang et al., 2018). TGFRβR2 is down regulated by a number of miRNAs, resulting in the progression of PCa. For example, miR-21 in positive feedback loop with AR downregulates TGFRβR2 and promotes PCa (Mishra et al., 2014). miR-212 has been reported to down regulate the expression of heterogenous nuclear ribonuclear protein H1 (hnRNPH1), a splicing protein vital for the growth and progression of PCa. Ectopic expression of miR-212 mimic directly modulated the expression of hnRNPH1 transrcipts which in turn reduced the expression of AR splice variant AR-V7 in PCa cells. The hnRNPH1 protein in conjunction with AR promotes the expression of steroid receptor coactivator-2 (SRC-3) vital for the activation of AR–regulated genes (Yang et al., 2016). Another miRNA named miR-2909 promotes PCa by interacting with 3′-UTR of sequence of TGFRβR2 and resulting in its downregulation. Moreover, overexpression of miR-2909 also results in decreased expression of SMAD3, further verifying its role in tumor progression (Ayub et al., 2017). Recent advances in the field of phytochemistry have begun to scratch the surface of molecular oncology. The natural compounds pose a wide range of therapeutic benefits that can help in culminating cancer. Interaction among miRNAs, natural compounds, and TGF-β signaling cascade is an emerging avenue for devising precision medicines for various cancers. The miRNAs and natural compounds can modulate the expression of TGF-β–associated signaling molecules.

Carnosol (CAR) is the main compound derived from the rosemary plant. It is a phenolic diterpene with strong antiproliferative ability both *in vitro* and *in vivo*. Data have suggested that carnosol can be implemented as a therapeutic option for the glioblastoma cells (Giacomelli et al., 2016). CAR can regulate a broad range of cellular processes affiliated with cancer proliferation, stemness, invasion, and metastasis through its interaction with key signaling pathways and miRNAs. Accumulating evidences have suggested that CAR has the ability to regulate the expression of miR-200c. miR-200c has been investigated for its role in modulation of TNF-α/TGF-β signaling and upregulated the expression of key downstream genes responsible for EMT (Snail, Slug, Twist, and ZEB1) *in vitro* (Giacomelli et al., 2017). Osthole is a natural coumarin obtained from the *Cnidium* plant. It has tremendous antiproliferative ability as it can reduce the tumor aggressiveness and metastasis. Osthole has the ability to suppress growth, metastasis, and EMT in PCa *via* modulating the expression of TGF-β/AkT/MAPK. Osthole-mediated downregulation of EMT promoter genes Snail, and miRNA-23a-3p triggers growth arrest and apoptosis in PCa cells (Wen et al., 2015). The data regarding the interplay between miRNA/TGF-β/natural compounds in PCa are scarce and require more research to be done. Table 1 shows the miRNAs and their interplay with TGF-β signaling and their effect on PCa status.

**TABLE 1:** List of miRNAs and TGFβ signaling to control PCa.

miRNA	Target	Effect on PCa	References
miR-486-5p	SMAD2	Upregulate	(Yang et al., 2017)
miR-505-3p	SMAD2 and SMAD3	Upregulate	(Tang et al., 2019)
miR-19a-3p	SMAD2 and SMAD4	Downregulate	(Wa et al., 2018)
miR-373-3p	TR4 and SMAD3	Downregulate	(Qiu et al., 2015)
miR-15a/16	pSMAD3	Upregulate	(Jin et al., 2018)
miR122/132	SOX	Downregulate	(Fu et al., 2016)
miR-34	SMAD3	Downregulate	(Fang et al., 2017)
miR1260b	SMAD4	Upregulate	(Hirata et al., 2014b)
miR301a	SMAD4	Upregulate	(Li et al., 2018c)
miR-205	SMAD4	Upregulate	(Zeng et al., 2016)
miR-93	TGFβR2	Upregulate	(Xu et al., 2018)
miR-133b	TGFβR1 and TGFβR2	Upregulate	(Huang et al., 2018)
miR-21	TGFβR2	Upregulate	(Mishra et al., 2014)
miR-2909	TGFβR2 and SMAD3	Upregulate	(Ayub et al., 2017)
miR-539	SMAD4 and DLX1	Downregulate	(Sun et al., 2019)
miR-582-3p and miR-582-5p	SMAD2, SMAD4, TGFβR1, and TGFβR2	Downregulate	(Huang et al., 2019)
miR-181a	TGFβR2	Upregulate	(Zhiping et al., 2017)
miR-221-5p	EMT (E-Cadherin, N-Cadherin, vimentin, Zinc finger homeobox 2 (ZEB2), SNAIL1/2, and TWIST	Downregulate	(Kiener et al., 2019)
miR-96	TGFβR2	Upregulate	(Siu et al., 2015)
miR-1 and miR-200	EMT (SLUG)	Downregulate	(Liu et al., 2013)
miR-183	SMAD4 and Dkk-3	Upregulate	(Ueno et al., 2013)
miR-485	Smurf-2 and TGFβR1	Downregulate	(Wang et al., 2018a)
miR-155	SMAD2	Downregulate	(Ji et al., 2014)

## Natural Compounds in PCa

The effectiveness of natural compounds in curbing various diseases including cancers has been proven experimentally (Salehi et al., 2019). In recent years, there are more attentions in elucidating therapeutic efficacy of natural compounds in PCa (Bayala et al., 2020; Zhang et al., 2020). These compounds target different pathways in cancer cells that are being exploited by such cells to ensure survival, growth, and also acquisition of metastatic capabilities. Inhibition of these pathways results in metastatic reversal, tumor growth regression, and apoptosis (Lajis et al., 2020) (Table 2).

**TABLE 2:** List of natural compounds, their sources, and pathway modulated in PCa.

Natural compound	Source	Pathway	References
Nobiletin	Citrus peels	TLR4 pathway	(Deveci Ozkan et al., 2020)
Curcumin	*Curcumin longa*	NF-Kb pathway and AR pathway	(Lajis et al., 2020)
Resveratrol	Grapes and berries	NF-Kb pathway	(Khusbu et al., 2020)
Daucosterol	*Crateva adansonii* DC	PI3K/Akt pathway	(Zingue et al., 2020)
Silibinin	*Silybum marianum*	PI3K/Akt pathway, ERK pathway, and JAK/STAT pathway	(Sherman et al., 2020)
Plectranthoic acid	*Ficus microcarpa*	TGF-β signaling and RAC1 signaling pathway	(Akhtar et al., 2018)
Osthole	*Cnidium monnieri*	TGF-β signaling and PI3k/AkT/mTOR pathway	(Wen et al., 2015)
Genistein	Soyabeans	TGF-β signaling, Smad4, and p38 MAPK	(Chen et al., 2018)
Oxymatrine	*Sophora japonica*	TGF-β signaling and Smad signaling	(Liu et al., 2016)
Tannic acid	Oak tree	TGF-β signaling, Smad signaling, SNAIL, and vimentin	(Pattarayan et al., 2018)
Paeoniflorin	*Paeonia lactiflora*	TGF-β signaling, Smad2/3 signaling inhibition, SNAIL, e-cadherin, and MMP-9 expression	(Wang et al., 2018b)

Natural compounds have been employed for the treatment of various human diseases for centuries. They have been found to be experimentally effective against different cancers. A plethora of studies have been conducted to delineate the complex interaction of natural compounds with molecular landscape of tumor cells both *in vitro* and *in vivo*. This has enabled researchers to determine novel compounds which can inhibit tumor growth, invasiveness, and metastasis. PCa is a complex disease orchestrated by a wide range of intrinsic and extrinsic factors. In PCa, the tumor growth is slow and has a persistently long latency period. These characteristics make PCa suitable for integration of natural compounds with other existing therapies for managing disease progression and mortality. The imbalance between abrupt cellular growth and apoptosis is the hallmark of PCa. Several oncogenes are overexpressed in PCa that lead to transformation of benign tumors to more aggressive metastatic PCa through suppression of the proapoptotic proteins. These changes trigger resistance to chemotherapy and radiotherapy. Natural compounds have been employed as adjuvants in combination with chemotherapy and radiotherapy to resensitize tumor cells toward treatment and also reduce drug resistance. There are a number of medicinal plants and their derivatives that have been reported to hold great therapeutic potential for PCa treatment. A phytochemical (amygdalin) present in the kernals of the member Rosaceae and prunasin has been found effective to reduce proliferation in PCa cell lines LnCaP and DU-145. Further insight into the tumor suppressor potential of amygdalin revealed that it reduced Bcl-2 and α6 integrin expression, and increased the cell cycle proteins (cyclin A, cyclin B, and cdk1) at G1-phase, resulting in the inhibition of growth, metastasis, adhesion, and chemotaxis (Saleem et al., 2018). Despite this, there are several cytotoxic effects caused by amygdalin. Leaf extracts of *Withania coagulans*, with anolides, were also reported to have antiproliferative, anti-migratory, and pro-apoptotic activities in DU-145 and PC-3 cells (Rehman et al., 2019). Caspase-dependent apoptosis is induced by ethanol extracts of *Hizikia fusiforme* in PC-3 cells where it downregulated c-Flip and promoted reactive oxygen species (ROS) production (Choi et al., 2020). Daucosterol obtained from *Crateva adansonii* has been reported to suppress growth, proliferation, and metastasis in LNCaP, DU-145, and PC-3 cell lines through upregulation of Bax protein and modulation of PI3K/AkT/mTOR pathway (Zingue et al., 2020). Daucosterol also phosphorylates JNK and elicits autophagy-induced apoptotic response (Gao et al., 2019). Phenols, coumaric acid and ascorbic acid, found in *Rosa canina*, bring about G1-phase growth arrest and induce intrinsic apoptosis by significantly reducing mitochondrial membrane potential (90%) and caspase-3 and caspase-7 activation in PC-3 cells (Kilinc et al., 2020). The extracts derived from *Lespedeza bicolor* induced G1-phase growth arrest *in vitro* inhibiting CDKs at the posttranscriptional or posttranslational level (Dyshlovoy et al., 2020). Cytotoxicity is one major stumbling block regarding the treatment of the PCa. Several natural compounds in combination with chemotherapy have been proven effective to reduce cytotoxicity and chemo-driven side effects. Licorice obtained from *Glycyrrhiza glabra* prevented tumor proliferation in PC-3 cell when administrated along with adriamycin (Gioti et al., 2020). Licorice also induced chemosensitivity in cisplatin-resistant DU-I45 and PC-3 cells (Martínez-Martínez et al., 2019). Docetaxel and thymoquinone reduced chemoprevention *in vitro*. A combination of the above reduced chemoresistance in C4-2B and DU-145 cells through modulation of PIK2/Akt axis (Singh et al., 2019). Neferine obtained from *Nelumbo nucifera* triggered apoptosis in DU-145 cell through enhancement of apoptosis *via* modulation of TRAIL and phosphorylation of JNK (Nazim et al., 2020). Excelsanone, an isoflavonoid found in the bark of *Erythrina excelsa*, has been tested for its anticancer properties in PCa cell lines DU-145 and PC-3 cells, and enhanced cytotoxicity (Gbaweng et al., 2020). Phytoalexin resveratrol, abundantly present in grapes and berries, halts EMT in PCa. It induces the lysosomal degradation of TRAF6 and indirectly suppresses NF-κB signaling and the transcription of SLUG which are among the main drivers of metastasis. Exposure of resveratrol in PC-3 and DU-145 cells reduced cell proliferation and viability (Khusbu et al., 2020). Another compound, ellagic acid, found in black raspberries prevented tumor growth in mice but at very high dose. Rest of the raspberry compounds such as protocatechuic acid and anthocyanin cyanidin-3-rutinoside did not have any recuperative effect on carcinogenesis both *in vivo* and *in vitro* (Eskra et al., 2020).

## Natural Compounds on the Basis of Androgen Status of the Cancer

The effectiveness of natural compounds is also evaluated on the basis of androgen status of the cancer. A nonpolar flavonoid, nobiletin, present in citrus peels has been reported to curb PCa growth by suppressing inflammation. The efficacy of its therapeutic influence is dependent on androgen status of PCa. Androgen-dependent LNCaP cell line is reported to be more sensitive to nobiletin than androgen-independent, metastatic PC-3 cell line. Mechanistically, it targets TLR4/TRIF/IRF3 and TLR9/IRF7 pathways by inhibiting TLR4, IRF3, TLR9, and IRF7 expression at the transcriptional level. Also, it reduces the mRNA and protein levels of IFN-α and IFN-β, which are downstream targets of TLR4 signaling cascade (Deveci Ozkan et al., 2020). Similarly, *Aegiceras corniculatum*–derived sakurasosaponin is reported as AR inhibitor. In sakurasosaponin-treated cell lines (LNCaP, C4-2, and 22Rv1), the rate of androgen receptor expression, along with few target genes (PSA, NKX3.1, and TMPRSS2), was decreased with increased dose and time. Furthermore, its treatment also induced intrinsic apoptosis by reducing mitochondrial membrane potential and Bcl-Lx expression. *In vivo* analysis revealed that it significantly attenuated tumor growth in AR-positive xenografted mice than in AR-negative xenografted mice (Song et al., 2020). Total saponins from *Paris forrestii* (PST3) constituting polyphillin D, dioscin, ophiopogonin C′, polyphyllin F, formosanin C, and glucopyranoside are isolated by Xia and team. They treated PC-3 and LNCaP cells with PST3 and found that it significantly reduced cell proliferation and promoted anti-invasiveness at minimum 1 μg/mL in LNCaP cells and 2 μg/mL in PC-3 cells. Its proapoptotic influence was in a dose-dependent manner in both cells lines (Xia et al., 2020). Resveratrol induces apoptosis in androgen-independent prostate cells by enhancing the expression of DUSP-1 which further suppresses NF-κB signaling and COX-2 expression (Martínez-Martínez et al., 2019). Curcumin is a widely studied natural compound in numerous cancers. In PCa, it induces cell proliferation inhibition, reversion of metastatic capability and cell death by inhibiting AR signaling *via* downregulating receptor transcription and translation or by inhibiting AR coactivators NF-κB, AP-1, and CBP. In LNCaP-xenografted mice (Tsui et al., 2008), its treatment halted signal transduction through AR *via* modulation of Wnt/β-catenin pathway (Hong et al., 2015; Lajis et al., 2020). In PC-3 cells, it decreased surface availability of AR by suppressing the expression of Hsp90 (Rivera et al., 2017) and promoted cell apoptosis through reducing mitochondrial membrane potential, promoting Bax expression and suppressing Bcl-2 expression (Yang et al., 2015). Synthetic derivatives of curcumin, cinnamaldehyde and dimethylamino derivative, were reported to improve sensitization of LNCaP cells for photodynamic therapy at concentration of 3 μM. Curcuminoid dimethylamino derivative reduced cell survivability in a dose-dependent manner (Kazantzis et al., 2020). Performing curcumin derivatives and photodynamic therapy together on other PCa cell lines and in animal models might give a novel method for curbing this disease. Triterpenes (trinordammaranolactone triterpene and dihydroxyoxodammarane triterpene) from *C. khorassanica* are reported effective against PCa, irrespective of hormone status (Sajjadi et al., 2020).

## Natural Compounds for the Treatment of Obesity Exposed PCa

Significance of natural compounds is also evaluated in obesity-exposed PCa cells. Studies have demonstrated that obesity promotes PCa proliferation and metastasis by inducing aberrant signaling through PI3K/Akt pathway, ERK pathway, and JAK/STAT pathway and by upregulating expression of pro-inflammatory COX-2 expression. Silibinin is a compound derived from *Silybum marianum*. Its *in vitro* treatment reported to cause reduction in signal transduction through all these pathways and downregulation of COX-2, leading to cell proliferation inhibition and reduced metastasis (Sherman et al., 2020). Silibinin is suggested as an effective therapeutic option for obese individuals suffering from PCa. Yet, the *in vivo* evidences are scarce to further validate its significance.

## Natural Compounds as Modulator of TGF Signaling in PCa

TGF-β signaling role in cancers is in dual manner: it acts both as oncogene and tumor suppressor. Very few investigations have focused on therapeutically targeting this pathway in PCa. Thus, understanding TGF-β pathway tumor suppressive or oncogenic role at different stages of cancer is very important (Colak and ten Dijke, 2017). In PCa, TGF-β pathway is aberrantly activated which mostly involves mutated downstream targets or mutation in TGF-β receptor (Seoane and Gomis, 2017; Grover et al., 2018). Many evidences are reported in which natural compounds acted as antagonist for this pathway in PCa. For instance, the dried powdered extract of *Ganoderma lucidum* inhibits angiogenesis of PC-3 cells. It induces this effect by hindering the phosphorylation of Akt and Erk1/2 which then fails to activate their downstream target AP-1, hence indirectly silencing the expression of TGF-β1 (Stanley et al., 2005). Resveratrol treatment to PCa cells also inhibits Akt activation by regulating miR-21 (Sheth et al., 2012), which can also lead to TGF-β1 expression downregulation and suppression of cancer cell proliferation. Compounds EGCG and myricetin halt TGF-β signaling in PC-3 cells by downregulating expression of TGFβR1 at the transcriptional level at a dose of ∼80 μM (Singh et al., 2016). TGFβR1 expression is reciprocal to the expression of miR-34c/b. In PC-3 cells, miR-34b/c expression is downregulated, which promotes TGFβR1 transcription and translation (Fang et al., 2017). It is possible that EGCG and myricetin might promote miR-34c/b expression that further inhibits TGFβR1posttranscriptionally. TGF-β and BMP pathway crosstalk is associated with PCa metastasis (Chen et al., 2012). Phenethyl isothiocyanate (PIT) treatment to PC-3 cells promoted miR-194 expression which downregulated BMP1 expression. PIT treatment also inhibited MMP2 and three expression which led to decreased invasiveness and metastatic capabilities (Zhang et al., 2016). Osthole, a bioactive coumarin, prevents EMT by simultaneously suppressing miR-23a-3p and TGF-β expression. TGF-β directly activates miR-23a-3p which then targets E-cadharin (Wen et al., 2015). Cairicoside E obtained from *Ipomoea cairica* has been investigated both *in vitro and in vivo*. It has been reported that Cairicoside E targets phosphorylation of Smads 2/3 triggered by TGF-β and prevents EMT in various tumors (Chen et al., 2017). Baicalin and baicalein are the two molecules that have been reported to modulate TGF-β signaling. These two compounds have been found effective both *in vitro* and *in vivo* in suppressing the proliferative potential via modulation of SLUG and NF-κβ signaling (Chung et al., 2015). Baicalin has also been demonstrated to inhibit the phosphorylation of Smads and promoted apoptosis and cell death *in vitro* (Zheng et al., 2016). Recent pharmacological evidences have shed light on paeoniflorin obtained from *Paenoia lactiflora* as a potential inhibitor of proliferation, metastasis, and invasion both *in vivo* and *in vitro.* In the mice model, it has been reported that paeoniflorin downregulated the expression of TGF-β, Snail, e-cadherin, vimentin, and MMP-9 (Ji et al., 2016) (Wang et al., 2018b). Altogether, downregulation of these molecules prevented proliferation and invasiveness in lung cancer. From these findings, it can be concluded that paeoniflorin could be a promising candidate to be tested for PCa. Evidence reported that tumor progression in early stages of PCa involves reduced signal transduction through TGF-β pathway. *Racemic gossypol*, present in cotton seeds, is reported to decrease cell proliferation and prolong the doubling time of PC-3 cells. Gossypol exerted its effects by promoting TGF-β1 expression at the transcriptional and translational levels (JIANG et al., 2004). Another compound, genistein inhibits invasiveness and promotes TGF-β signaling by suppressing onco-miR-1260b expression and removing smad4 from its regulatory control. Genistein also induces epigenetic modification (DNA demethylation) of smad4 gene to promote its transcription (Hirata et al., 2014a). Its treatment also induces miR-574-3p (tumor suppressor miRNA) expression (Chiyomaru et al., 2013), but its association with TGF-β signaling is yet to be determined. Berberine, a natural compound obtained from barberry, has been reported to have extensive antiproliferative, anti-microbial, and anti-inflammatory properties (Tillhon et al., 2012). The role of berberine as a potential inhibitor of metastasis has been well documented. A study conducted by Kou et al. demonstrated that berberine prevented cell adhesion *via* modulating the expression of E-cadherin, vimentin, and fibronectin in cell lines. Berberine downregulates the expression of several EMT genes *via* regulating the PI3K/AkT signaling axis and retinoic acid receptor signaling in various cancers (Kou et al., 2016). Arctigenin is another natural liganin obtained from the plants of Asteraceae family. Arctigenin possesses tremendous antiviral and antiproliferative properties *in vitro*. Recent studies have demonstrated that arctigenin modulated the expression of TGF-β expression and inhibited the phosphorylation of the Smad2/3, and thus prevented the downstream activation of EMT in lung cancer cell lines. Moreover, overexpression of E-cadherin in a dose-dependent manner increased the expression of ERK and *ß*-catenin that in turn facilitated the expression of TGF-β and triggered EMT (Xu et al., 2017). All together, these findings shed light that arctigenin may be implemented as a potential therapeutic approach for culminating PCa cancer as well. Alpha-solanine is another natural compound that has been reported to prevent invasiveness, EMT, and proliferation of the PCa *via* modulation of ERK and PI3K/Akt axis. Alpha-solanine prevents phosphorylation of these molecules that in turn prevents the activation of EMT target genes and thus inhibit growth of PCa cell *in vitro* (Shen et al., 2014). Plectranthoic acid (PA) has been investigated for its role in inhibiting cell proliferation and invasiveness in PCa *via* modulation of the TGF-β signaling. Recent findings suggest that PA triggers growth arrest and inhibition of metastasis *via* regulation of RAC1 signaling (Akhtar et al., 2018). A phytochemical obtained from *Solanum nigrum Linn* (α-solanum) has been found active against TGF-β–mediated EMT transition in PCa cells *in vitro*. α-solanum suppressed MMP expression *via* modulation of ERK/AkT axis and thus prevented EMT in PCa (Shen et al., 2014). Sulforaphane is a natural compound found abundant in broccoli and other cruciferous vegetables. This phytochemical has tremendous anti-oxidant properties. Sulforaphane has been documented to prevent growth and proliferation in various tumors including PCa. It directly targets TGF-β signaling and *via* suppression of SLUG (Amjad et al., 2015). Sulforaphane has also been shown to prevent metastasis through interplay among miR-616-5p, beta-catenin, and GSK-3β signaling cascade (Wang et al., 2017). Osthole derived from *Cnidium monnieri* has been found effective in the prevention of metastasis and invasion in different cancers. Osthole blocks EMT *via* modulation/suppression of TGF-β/AkT/MAPK axis, downregulation of Snail, and upregulation of E-cadherin both *in vitro* and *in vivo* (Wen et al., 2015). However, few compounds discussed above have potential to curb PCa progression, but none of these compounds are validated in animal models. Also, the influence of these compounds according to androgen-dependent or independent status also needs to be determined. Finally, with progressing cancer status of TGF-β pathway activation changes, so with further understanding of actors involved in switching on/off in this pathway, the development of natural compound-based therapy is warrant to pursue.

## Perspectives

PCa is a serious anomaly that affects male population in the world. A number of studies over the years provide strong evidence of the involvement of both TGF-β signaling and miRNAs in the development and progression of PCa. The interplay between miRNAs and TGF-β signaling is so crucial that it may aid in resolving the dual behavior of TGF-β signaling in various cancers. miRNAs have a dual role in regulating TGF-β signaling. They can either modulate the expression of the key components of TGF-β signaling machinery such as the receptors, SMADs, and antiapoptotic behavior of the TGF-β–mediated oncogenes. This results in decreased cellular growth and metastasis. However, during proliferation of PCa, expression of these tiny molecules is downregulated, causing direct activation of downstream TGF-β effectors. The miRNAs can also directly act on genes which are activated by TGF-β signaling, thus either inhibiting or promoting the proliferation of PCa. The oncogenic miRNAs are upregulated in highly metastatic PCa cells. They achieve this by downregulating the expression of tumor suppressor miRNAs in a variety of ways leading to cancer progression. From these findings, it can be observed that the switching of miRNAs expression is a pivotal mechanism to understand the dual role of TGF-β signaling in PCa. TGF-β signaling is renowned for regulating molecular landscape of tumor cells and immune responses. Therefore, understanding the interplay between miRNAs and TGF-β signaling could help in the development of diagnostic and prognostic biomarkers. In addition, the interplay between miRNAs and TGF-β signaling is emerging as platform for therapeutic interventions. This will lead to the inclusion of miRNAs that specifically target TGF-β signaling pathway in to clinical trials for the new therapeutic intervention in PCa. Given the prospect, miRNA-mediated regulation of TGF-β signaling in PCa may be promising for the treatment of aggressive prostate tumors.

Phytochemicals have recently emerged as a promising field that can help in prevention and even treatment of PCa. It has been reported that several medicinal plants, herbs, and phytochemicals have tremendous potential in preventing various cancers. The chemical constituents of various plant species have a preponderant role in the production of bioactive phytochemicals. The phenolic extracts of several plants prevented the proliferative potential of the PCa followed by alkaloids and terepnoids. Despite these encouraging results, only few of the phytochemicals have undergone clinical trials. More clinical evidences are required to validate the *in vitro* and *in vivo* studies conducted on natural compounds and their interplay with miRNAs and TGF-β signaling in PCa. Combining natural compounds, miRNAs and TGF-β signaling will ensure better chemoprevention and advanced therapeutic strategies for PCa.

## Author Contributions

Conceptualization: JS-R, ZJ, KK, AR, AS, SR, BS, WC, WK, WK-K, AG-L, and PH. Validation investigation, resources, data curation, writing: all authors. Review and editing: WC, JS-R, WK, and BS. All authors contributed to the article and approved the submitted version.

## Conflict of Interest

The authors declare that the research was conducted in the absence of any commercial or financial relationships that could be construed as a potential conflict of interest.
